# The complete mitochondrial genome of the stick tea thrips *Dendrothrips minowai* (Thysanoptera: Thripidae)

**DOI:** 10.1080/23802359.2017.1419099

**Published:** 2017-12-26

**Authors:** Shi-Chun Chen, Hong-Yan Jiang, Ping Peng, Xiang Hu, Qiang Lin, Xiao-Qing Wang

**Affiliations:** Tea Research Institute of Chongqing Academy of Agricultural Science, Chongqing, P. R. China

**Keywords:** Mitochondrial genome, Dendrothrips minowai, Thysanoptera, tea pest

## Abstract

The stick tea thrips, *Dendrothrips minowai* Priesner (Thysanoptera: Thripidae), is a major pest of tea plantation and poses a considerable economic threat to tea industry. The mitochondrial genome of *D. minowai* have been sequenced and annotated completely. The entire genome is 14,631 bp in length with an A + T content of 78.53% (GenBank accession No. MF582634). The stick tea thrips mt genome encodes all 37 genes that are typically found in animal mt genomes, consists of 13 protein-coding genes, two ribosomal RNA genes, and 22 transfer RNA genes. The gene order is unique and different from that of the other thrips. The A + T-rich region is 149 bp long and contains two poly-T stretchs. Phylogenetic analysis was performed using 13 protein-coding genes with six thrips showed that *D. minowai* and other five Thripidae species were clustered into a branch, which is formed a sister clade to *H. aculeatus* (family Phlaeothripidae).

The stick tea thrips, *Dendrothrips minowai* Priesner (Thysanoptera: Thripidae), is a major pest of tea. Adults and nymphs preferentially feed on tender leaves using rasping–sucking mouth parts, which cause infectious diseases in tea plants and seriously impact tea quality and yield (Bian et al. 2016). In this study, the stick tea thrips samples were collected from the tea plantation at Yongchuan, Chongqing, China in 2016, subsequently identified to species by morphology. Voucher specimens (#CQNKY-TH-01-01-01) were deposited at the Insect Collection, Tea Research Institute of Chongqing Academy of Agricultural Science, Chongqing, China.

The complete mitochondrial genome of the stick tea thrips is a typical closed-circular DNA molecule of 14,631 bp in length (GenBank accession No. MF582634). The mt genome of *D. minowai* is the second smallest mt genome among all sequenced Thysanoptera species and larger than *Haplothrips aculeatus* (14,616 bp). The total nucleotide composition of the J-strand of the mt genome as follows: A = 40.93% (5988), C = 11.63% (1702), G = 9.84% (1440), and T = 37.60% (5501), with a total A + T content of 78.53%, that is heavily biased toward A and T nucleotides. AT- and GC-skew of the whole J-strand of *D. minowai* is 0.042 and −0.083, respectively.

The mt genome of *D. minowai* encodes all 37 genes usually found in animal mt genomes, including 13 protein-coding genes (PCG), two ribosomal RNA genes, and 22 transfer RNA genes. Gene rearrangement occurred frequently among order Thysanoptera, and the *Scirtothrips dorsalis* South Asia 1 species has a genome consisting of two circular chromosomes (Dickey et al. 2015). The mt gene arrangement in *D. minowai* differs from that of the other thrips. In the mt genome of *D. minowai*, a total of 29 bp overlaps have been found at eight gene junctions. The mt genome has a total of 309 bp intergenic sequence without the putative A + T-rich region. The intergenic sequences are at 16 locations ranging from 1 to 76 bp, the longest one locates between *trnL_1_* and *nad4L*. In order Thysanoptera, mt genomes of thrips usually contain two or more putative control regions (Shao et al. 2001; Yan et al. 2012; Yan et al. 2014; Dickey et al. 2015). However, there is only one putative control region have been found in the mt genome of *D. minowai*. The putative control region of this genome is 149 bp long and located between the *trnS_1_* and *nad5* genes. The A + T content of this region is 91.95%, the highest level of each region in this mt genome. This region contains two poly-T with 23 bp and 21 bp in length, respectively.

All 22 tRNA genes usually found in the mt genomes of insects have been identified in *D. minowai*, 18 tRNA genes are encoded by the J-strand and the other four genes are encoded by the N-strand. The nucleotide length of tRNA genes is ranging from 55 bp (*trnS_1_*) to 74 bp (*trnV*), and A + T content is ranging from 66.67% (*trnI*) to 89.23% (*trnS_2_*). Like most insect, 21 tRNA genes have cloverleaf shaped secondary structure and *trnS_1_* gene lacks the dihydrouridine (DHU) arm. The two rRNA genes have been identified on the J-strand in the mt genome: the *rrnL* gene locates between *trnV* and *trnS_2_*, and the *rrnS* gene between the *trnF* and *atp8*. The length of *rrnL* and *rrnS* is 1092 bp and 750 bp, and their A + T content is 79.30% and 80.00%, respectively.

The total length of all 13 protein-coding genes is 10,932 bp, which is accounting for 74.72% of the whole genome sequence. The A + T content of the 13 genes ranges from 71.35% (*cox1*) to 84.14% (*nad6*). All of the 13 PCGs start with ATN codons, ATT for *atp8*, *cox1*, *cob*, *nad1* and *nad4* and ATA for the remainder genes. Two genes, *cob* and *nad2*, have incomplete terminal codons consisting of single T nucleotide, and the other PCGs stop with TAA or TAG. The incomplete stop codon T is commonly reported and could produce functional stop codons in polycistronic transcription cleavage and polyadenylation mechanisms (Boore 2001). We analyzed the amino acid sequences of 13 PCGs with maximum likelihood (ML) method to learn the phylogenetic relationship of *D. minowai* with other thrips. The mt genome sequence of *Drosophila melanogaster* (GenBank accession no. DMU37541) was used as an outgroup. In the tree, *D. minowai* and other five Thripidae species were clustered into a branch (Figure 1), which is formed a sister clade to *H. aculeatus* (family Phlaeothripidae). It infers that the stick tea thrips is closely related to species of Thysanoptera.

**Figure 1.**
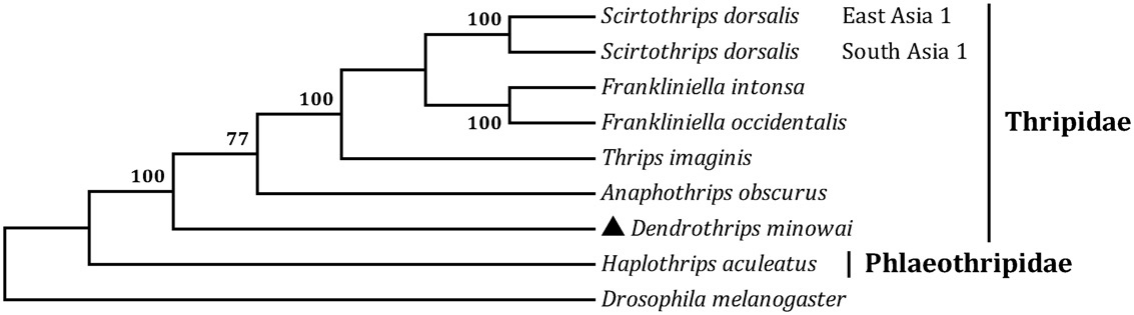
The maximum likelihood (ML) phylogenetic tree of *Dendrothrips minowai* and other moths. The GenBank accession numbers used for tree constructed are as follows: *Anaphothrips obscurus* (KY498001), *Frankliniella intonsa* (JQ917403), *Frankliniella occidentalis* (JN835456), *Haplothrips aculeatus* (KP198620), *Scirtothrips dorsalis* East Asia 1 (KM349826), *Scirtothrips dorsalis* South Asia 1 (KM349827 and KM349828), and *Thrips imagines* (AF335993).
